# Supplementary material for: Single Cell Transcriptomic and Chromatin Profiles Suggest Layer Vb Is the Only Layer With Shared Excitatory Cell Types in the Medial and Lateral Entorhinal Cortex
Source: Front Neural Circuits. 2022 Jan 26;15:806154. doi: 10.3389/fncir.2021.806154 (PMC8826650; doi:10.3389/fncir.2021.806154)
Supplement: Supplementary file 14 [file Data_Sheet_1.ZIP › Sup_vs_Deep_Motifs/homerResults/motif2.similar.html]

motif2

## Information for motif2

A
G
C
T
C
G
A
T
C
T
A
G
A
C
G
T
A
G
C
T
A
C
G
T
C
T
G
A
A
G
C
T
G
T
C
A
G
C
A
T
  
Reverse Opposite:  

C
G
T
A
C
A
G
T
T
C
G
A
G
A
C
T
C
G
T
A
T
C
G
A
C
G
T
A
A
G
T
C
C
G
T
A
T
C
G
A
  

|  |  |
| --- | --- |
| p-value: | 1e-326 |
| log p-value: | -7.508e+02 |
| Information Content per bp: | 1.636 |
| Number of Target Sequences with motif | 14247.0 |
| Percentage of Target Sequences with motif | 42.52% |
| Number of Background Sequences with motif | 2767.3 |
| Percentage of Background Sequences with motif | 32.42% |
| Average Position of motif in Targets | 333.4 +/- 242.3bp |
| Average Position of motif in Background | 373.1 +/- 264.0bp |
| Strand Bias (log2 ratio + to - strand density) | 0.1 |
| Multiplicity (# of sites on avg that occur together) | 1.30 |
| Motif File: | file (matrix) reverse opposite |

### Similar de novo motifs found

|  |  |  |  |  |  |  |  |
| --- | --- | --- | --- | --- | --- | --- | --- |
| Rank | Match Score | Redundant Motif | P-value | log P-value | % of Targets | % of Background | Motif file |
| 1 | 0.647 | G A C T G T C A G C T A C G A T C A G T C T G A T C A G T C A G G C T A G T C A | 1e-264 | -609.467471 | 35.17% | 26.52% | motif file (matrix) |
| 2 | 0.844 | C A G T A C G T A C G T C T A G A C G T A C G T A C G T C G A T | 1e-258 | -595.363492 | 53.06% | 43.70% | motif file (matrix) |
| 3 | 0.630 | G A C T C G T A G C T A C G A T A C G T C T G A T C A G T G A C | 1e-241 | -555.182380 | 22.70% | 15.75% | motif file (matrix) |
| 4 | 0.884 | A G T C C G A T C T G A A G C T C G A T A C G T C G T A G A T C | 1e-210 | -485.821957 | 49.35% | 40.95% | motif file (matrix) |
| 5 | 0.776 | G C A T G T C A C G T A C G T A C G T A C G T A A G C T C G T A C G T A C G T A G C T A G T C A | 1e-173 | -399.492314 | 25.57% | 19.29% | motif file (matrix) |
| 6 | 0.675 | C A G T T A C G G A C T C G A T A G C T C G A T A C T G C G A T A C G T C A G T | 1e-140 | -322.549652 | 19.96% | 14.85% | motif file (matrix) |
| 7 | 0.669 | G A T C C G A T T C G A C G A T A C G T C G T A C T G A C A G T C T G A A T C G | 1e-137 | -317.685996 | 52.36% | 45.52% | motif file (matrix) |
| 8 | 0.799 | G A C T A C G T T C A G A C G T A C G T C A T G A C T G A G C T | 1e-124 | -287.724456 | 58.74% | 52.26% | motif file (matrix) |
| 9 | 0.676 | C T A G G A C T C G A T C T A G A G C T C G A T C A T G G C A T A G C T C A G T | 1e-99 | -229.547791 | 15.84% | 11.92% | motif file (matrix) |
| 10 | 0.851 | A C G T A C G T A C T G A C G T A C G T A C G T C G T A A G T C | 1e-94 | -217.930367 | 2.91% | 1.40% | motif file (matrix) |
| 11 | 0.729 | C T A G C A G T C G T A G T C A C T G A G A T C G C T A G T C A G C A T C A T G G T A C G T C A | 1e-85 | -195.931332 | 0.27% | 0.02% | motif file (matrix) |
| 12 | 0.681 | C T G A T C G A A G T C G C A T C G T A T C G A G T A C G T C A G C A T C G T A | 1e-84 | -195.017457 | 19.77% | 15.76% | motif file (matrix) |
| 13 | 0.617 | C A G T C G A T G C A T C G T A G C A T C T G A G C T A C G T A G C A T C G T A G A C T C G T A | 1e-29 | -67.014329 | 5.19% | 3.94% | motif file (matrix) |
| 14 | 0.679 | C T G A C G A T C T G A A C G T C G A T G C A T G C T A A C G T | 1e-26 | -60.798734 | 11.33% | 9.56% | motif file (matrix) |
| 15 | 0.663 | C G T A A G T C C G T A C G T A C G T A A G T C C G T A A C T G C G T A A C T G | 1e-23 | -54.796437 | 0.51% | 0.22% | motif file (matrix) |
| 16 | 0.695 | A C G T A C T G A C G T A C G T A G C T A C T G A C G T G T A C C G T A A C T G | 1e-22 | -51.694545 | 0.58% | 0.27% | motif file (matrix) |
| 17 | 0.607 | C G T A A C G T A C G T C G T A C G T A A C G T A C T G A C G T A C G T A C G T | 1e-16 | -37.943103 | 0.33% | 0.13% | motif file (matrix) |
